# Herbal and Natural Products for Antibiotic-Associated Diarrhea: A Systematic Review of Animal Studies Focusing on Molecular Microbiome and Barrier Outcomes

**DOI:** 10.3390/ph19010064

**Published:** 2025-12-29

**Authors:** Ji Hye Hwang, You-Kyoung Choi

**Affiliations:** 1 Department of Acupuncture & Moxibustion Medicine, College of Korean Medicine, Gachon University, Seongnam 13120, Republic of Korea; jhbori@nate.com; 2 Department of Korean Internal Medicine, College of Korean Medicine, Gachon University, Seongnam 13120, Republic of Korea

**Keywords:** antibiotic-associated diarrhea, herbal medicine, natural products, microbiome, short-chain fatty acids, epithelial barrier, animal models, systematic review

## Abstract

**Background/Objectives:** Antibiotic-associated diarrhea (AAD) arises from antibiotic-induced disruption of microbial diversity, metabolic activity, epithelial integrity, and mucosal immunity. Probiotics are widely used but often show limited efficacy under antibiotic pressure. Herbal and natural products (HNPs) may provide multi-target benefits by modulating microbiota-dependent and host-directed pathways. This review synthesized animal studies evaluating HNP or HNP–probiotic combination (HNP–C) therapies using molecular microbiome endpoints. **Methods:** Following PRISMA 2020 guidelines, controlled in vivo studies assessing HNP or HNP–C interventions for AAD were searched in Pubmed, EMBASE, Web of Science, Scopus, and CNKI through November 2025. Eligible studies reported microbial diversity, taxonomic shifts, short-chain fatty acids (SCFAs), barrier markers, or immune responses. Risk of bias was assessed using the SYRCLE tool. Due to heterogeneity, findings were narratively synthesized. **Results:** Twenty-seven studies met inclusion criteria (21 HNP, 6 HNP–C). HNP monotherapies consistently improved α-diversity, shifted β-diversity toward healthy controls, restored SCFA-producing taxa, and increased SCFA levels. They also enhanced tight junction proteins and reduced inflammatory cytokines. HNP–C interventions demonstrated more comprehensive microbial, epithelial, and immune recovery; however, only two studies included direct comparisons among HNP-only, probiotic-only, and combination groups. In these, HNP–C showed greater improvements than individual components, suggesting complementary or potentially complementary or additive effects. Other HNP–C studies were limited by absent comparator arms. **Conclusions:** HNPs appear to support recovery of microbial diversity, metabolic function, epithelial barrier integrity, and immune regulation by engaging microbiota-dependent and host-mediated mechanisms. HNP–C strategies may offer complementary benefits, although rigorously controlled comparative studies and clinical validation remain needed.

## 1. Introduction

Antibiotic-associated diarrhea (AAD) is a common and clinically significant adverse effect of antibiotic therapy, affecting 5% to over 30% of patients depending on individual susceptibility [1,2]. Beyond causing transient gastrointestinal symptoms, AAD disrupts intestinal homeostasis, reduces treatment compliance, and increases the risk of recurrent infection [3,4]. Broad-spectrum antibiotics produce abrupt shifts in the gut microbiota by rapidly reducing microbial abundance and diversity, which weakens colonic resistance [5]. They also inhibit short-chain fatty acid (SCFA) production and compromise tight junction integrity between epithelial cells [4,6,7]. These disturbances increase intestinal permeability, heighten inflammatory responses, and create conditions that favor opportunistic pathogens [8]. Importantly, microbiota disruption can persist for months after antibiotic cessation, indicating delayed or incomplete ecological recovery [9] and highlighting the importance of microbial stability and functional redundancy in host protection [10].

Conventional management strategies, including probiotics, dietary modifications, and symptomatic therapies, show variable effectiveness. Although probiotics replenish beneficial microbiota, they generally colonize the gut only transiently and inconsistently during or after antibiotic treatment [11,12]. Prebiotics promote the growth of commensal bacteria but have limited capacity to directly restore the epithelial barrier or immune function [12]. Growing interest has therefore focused on herbs and natural products (HNPs), which target the microbiota while simultaneously modulating multiple pathological dimensions, including microbiota structure, SCFA metabolism, barrier integrity, and mucosal immunity [13,14,15,16]. For example, polysaccharides supply selectively fermentable substrates that enhance SCFA production and support the recovery of symbiotic bacteria, whereas flavonoids and saponins modulate host inflammatory pathways such as NF-κB and MAPK and contribute to restoring tight junctions [14,16].

Several herbal medicines, including *Zingiber officinale*, Shenlingbaizhusan, and Xianglian Pill, have been evaluated in animal models of AAD [17,18,19], but none included microbiome sequencing results. This underscores the importance of incorporating microbiological endpoints when assessing therapies for microbiome-driven diseases. Despite growing interest in herbal treatments targeting the microbiota, many studies, including preclinical evaluations of gut-protective effects, do not elucidate underlying microbiological mechanisms by integrating molecular microbiome profiling such as 16S rRNA sequencing [13,14,17]. This knowledge gap is also reflected in existing literature reviews. For instance, a pediatric meta-analysis of AAD primarily focused on clinical endpoints without assessing the microbiome [20]. Likewise, a recent narrative review summarized the role of herbal polysaccharides in chronic diarrhea phenotypes, including irritable bowel syndrome with diarrhea (IBS-D), osmotic diarrhea, bile acid diarrhea, and AAD, but did not systematically integrate controlled in vivo AAD studies that included microbiome sequencing [21]. Similarly, recent systematic reviews of herbal medicines for AAD have focused on clinical outcomes, with limited microbiome endpoints [22].

Therefore, despite growing evidence that HNPs can modulate the gut microbiota, a synthesis focusing on controlled AAD animal studies integrating microbiological, metabolic, and barrier–immune endpoints remains lacking. To address this gap, this systematic review limits its scope to single-component HNPs and simple herbal medicine–probiotic combinations, excluding multi-component formulations (more than three components) to maintain mechanistic clarity. The aim of this review was to develop an integrated mechanistic framework by comparing the effects of AAD therapies on microbial diversity, β-diversity, SCFA production, epithelial barrier integrity, and mucosal immune regulation from a microbiota-centered perspective.

## 2. Materials and Methods

### 2.1. Study Design

This systematic review was conducted according to PRISMA 2020 guidelines [23], with the completed PRISMA checklist provided in (Supplementary Material Table S1). The review focused exclusively on controlled in vivo animal experiments evaluating herbal or natural product interventions for AAD and reporting quantitative gut microbiota outcomes. This systematic review was prospectively registered in PROSPERO (CRD420251136553) https://www.crd.york.ac.uk/PROSPERO/view/CRD420251136553 (accessed on 23 December 2025).

### 2.2. Eligibility Criteria

This review included animal models of AAD with no restrictions on species, gender, age, or weight. Interventions were categorized into two main groups: HNP, representing single-agent herbal or natural product interventions, and HNP-C, representing combination approaches involving co-administration with probiotics or synbiotics. Multi-component herbal formulations containing three or more ingredients were excluded from the main synthesis because of compositional heterogeneity and will be addressed in a separate review. Comparative groups included drug vehicles, untreated controls, antibiotic monotherapy, or established positive controls (e.g., probiotics).

The primary outcomes focused on gut microbiota composition assessed by 16S rRNA sequencing or metagenomic profiling, as well as other molecular approaches (e.g., DGGE, PCR-DGGE, or targeted qPCR), provided that microbial composition or taxonomic shifts were reported, including α-diversity, β-diversity, and taxonomic changes. Secondary outcomes included SCFAs, bile acids, tight junction proteins (e.g., ZO-1, occludin, claudin-1), mucins, SIgA, cytokines, and markers of systemic or mucosal inflammation.

Included study types were limited to controlled in vivo animal studies published in peer-reviewed journals. Exclusion criteria consisted of literature reviews, study protocols, in vitro studies, non-AAD models, probiotic monotherapy interventions, and studies lacking extractable microbiome data.

### 2.3. Information Sources

A comprehensive search of major international databases PubMed/MEDLINE, EMBASE, Web of Science, Scopus, and CNKI (China National Knowledge Infrastructure) was conducted from database inception to November 2025. An additional search of domestic medical databases, including OASIS, KISS, and KMbase, was performed but yielded no further eligible studies. No language restrictions were applied to ensure maximal capture of relevant preclinical evidence.

### 2.4. Search Strategy

The search strategy incorporated both controlled vocabulary terms (e.g., MeSH) and free-text keywords related to the following:

- antibiotic-associated diarrhea
- antibiotic-induced dysbiosis
- herbal medicine
- natural product
- polysaccharide
- specific herbal agents
- gut microbiota
- microbiome
- 16S rRNA
- animal model

Equivalent Chinese terms were applied for CNKI searches (Subject Term).

Full database-specific search strings will be provided in Table S1.

### 2.5. Study Selection

All retrieved records were consolidated and deduplicated before screening. Two reviewers independently screened titles and abstracts, followed by full-text evaluation according to predefined eligibility criteria. Discrepancies were resolved through discussion. 

### 2.6. Data Extraction

A standardized extraction form was developed. Extracted variables included study characteristics, animal details, antibiotic regimen, intervention type (HNP or HNP-C), microbiome assessment method, α- and β-diversity indices, key taxonomic changes, SCFAs, barrier markers, immune mediators, main findings, and study limitations. Numeric data were extracted directly, while graphical data were collected only when values were clearly interpretable.

### 2.7. Risk of Bias Assessment

Risk of bias was evaluated independently by two reviewers using the SYRCLE tool [24], with disagreements resolved by consensus.

### 2.8. Data Synthesis

Due to substantial methodological heterogeneity across animal models, antibiotic protocols, intervention types, and microbiome analytic platforms, a quantitative meta-analysis was not planned. Instead, findings were synthesized narratively, with comparative mapping of outcomes between HNP and HNP-C interventions.

## 3. Results

### 3.1. Study Selection

**Figure 1:**
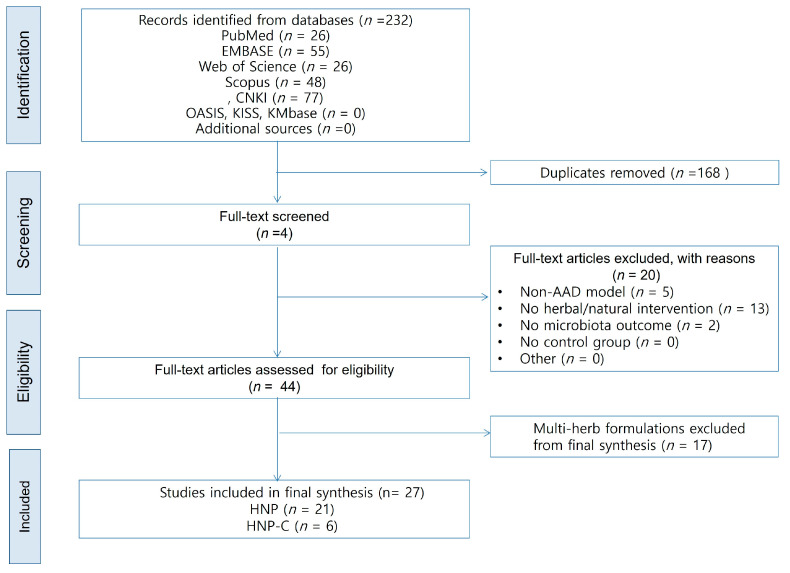
PRISMA 2020 flow diagram of study selection. HNP = Herbal/Natural Product; HNP-C = Herbal/Natural Product Combination.

A total of 232 records were identified through searches of the PubMed, EMBASE, Web of Science, Scopus, and CNKI databases. After removing 168 duplicates, 64 full-text articles were assessed for eligibility. Twenty studies were excluded: five used non-AAD models, 13 did not evaluate herbal or natural product interventions, and two lacked extractable microbiome outcomes. Ultimately, 44 studies met the initial inclusion criteria. Of these, 17 multi-component prescription studies were excluded from the primary synthesis because of compositional heterogeneity. The final analysis, therefore, included 21 HNP interventions [25,26,27,28,29,30,31,32,33,34,35,36,37,38,39,40,41,42,43,44,45] and six HNP-combination (HNP–C) interventions [46,47,48,49,50,51], yielding a total of 27 studies. The study selection process is depicted in the PRISMA flow diagram (Figure 1).

### 3.2. Study Characteristics

A total of 27 animal studies were included in the final synthesis, comprising 21 HNP interventions [25,26,27,28,29,30,31,32,33,34,35,36,37,38,39,40,41,42,43,44,45] and six HNP-C interventions [46,47,48,49,50,51]. Most investigations used C57BL/6 or BALB/c mice (81%), while the remaining studies employed Sprague–Dawley rats. Reporting of sex and age was inconsistent across studies.

Among HNP interventions, polysaccharides were the most frequently studied category. These were derived from *Astragalus membranaceus* [30], *Panax* species [26,28,45], *Poria cocos* [31,36,48], edible fungi or marine sources [32,37,38], *Dioscorea opposita* and related tubers [29,34,35], and plant-derived foods such as bamboo shoot [39] and purple sweet potato [33]. Tangeretin was the primary flavonoid investigated [25], while extracts or gingerols from *Zingiber officinale* represented the major essential-oil components [17,43,44]. Saponin-based interventions were mainly derived from *Cistanche deserticola* [41], and blueberry leaf polyphenols were also evaluated as a distinct HNP extract [40].

HNP-C interventions combined probiotics with herbal or natural bioactive compounds, including Panax-derived polysaccharides [46], *Astragalus* polysaccharides [47], *Poria cocos* polysaccharide preparations [48], bioactive glycans [49], medicinal minerals such as maifan stone [50], and chitosan oligosaccharides [51], to enhance microbiota restoration.

Most studies assessed gut microbiota composition using 16S rRNA sequencing; however, several studies employed lower-resolution community profiling methods such as DGGE or PCR-DGGE, and one HNP–C study used targeted qPCR without 16S profiling. SCFAs were reported in 17 studies (63%), tight junction or mucin markers in 15 studies (56%), and immune-inflammatory markers in 18 studies (67%).

Owing to heterogeneity in antibiotic regimens, animal strains, and analytic platforms, findings were synthesized narratively rather than through meta-analysis. Detailed study characteristics are presented in Table 1.

### 3.3. Microbial Diversity and Composition (Table 2 and Table 3)

Comparative summary of therapeutic effects between herbal/natural product (HNP) monotherapy (*n* = 21) and HNP-combination (HNP-C) interventions (*n* = 6) in antibiotic-associated diarrhea models. Reporting frequencies indicate the number of studies that assessed each endpoint.

#### 3.3.1. Effects on α-Diversity

Of the 21 HNP studies, 20 quantitatively assessed α-diversity (Shannon, Simpson, Chao1, ACE) [25,26,27,28,29,30,31,32,33,34,35,36,37,38,39,40,41,42,43,44,45], all demonstrating recovery of richness and evenness suppressed by antibiotics. One study used DGGE without numerical indices [30]. All six HNP-C studies also reported increased α-diversity [46,47,48,49,50,51]; five included quantitative metrics [46,47,48,49,51], whereas one relied on qPCR-based assessment only [50]. Notably, only two studies incorporated herbal-only and probiotic-only comparator arms [47,48], both showing faster or more complete α-diversity restoration with combination therapy. Overall, both HNP and HNP-C interventions reversed antibiotic-induced reductions in α-diversity, with synergistic effects of HNP-C supported exclusively by comparator-controlled studies.

#### 3.3.2. Effects on β-Diversity

Eighteen HNP studies reported β-diversity outcomes using PCoA or NMDS [25,26,27,28,29,31,32,33,35,36,37,38,39,40,41,42,43,44,45], all showing shifts toward the healthy control cluster, although complete normalization was not consistently observed. Three studies provided qualitative clustering results only [30,34].

All six HNP-C studies similarly demonstrated convergence toward the control cluster [46,47,48,49,50,51]. Among these, two studies that included appropriate comparator arms [47,48] reported more pronounced β-diversity shifts with combination therapy.

Other HNP-C studies showed consistent directional trends but lacked the design elements necessary to confirm additive effects. Overall, both intervention types facilitated ecological recovery, with more robust evidence of microbiota remodeling emerging from the limited comparator-designed HNP-C studies.

#### 3.3.3. Key Taxonomic Shifts

Across both intervention categories, consistent taxonomic patterns were observed: enrichment of *Lactobacillus*, *Bifidobacterium*, Muribaculaceae, Lachnospiraceae, and Ruminococcaceae, alongside suppression of *Escherichia–Shigella*, Enterobacteriaceae, and *Clostridium sensu stricto* [25,26,27,28,29,30,31,32,33,34,35,36,37,38,39,40,41,42,43,44,45,46,47,48,49,50,51]. Only two HNP-C studies with comparator arms [47,48] showed greater restoration of beneficial taxa and larger reductions in Proteobacteria-associated taxa compared with monotherapy. Overall, both HNP and HNP-C interventions restored commensal abundance, although mechanistic conclusions favoring HNP-C remain restricted to studies with robust comparator designs.

**Table 2:** Effects of HNP and HNP-C on Gut Microbiota and Host Outcomes.

Group	Study ID	Intervention (Short)	α-Diversity Effect	β-Diversity Effect	Key Taxa Changes	SCFA	Barrier Markers	Immune and Inflammatory Markers	Histopathology	Mechanistic Notes
HNP	Xu et al. [25]	Tangeretin (Citrus flavonoid)	↑ Diversity (recovery trend)	PCoA shifted toward NC	↑ Lachnospiraceae, Bacteroidaceae; ↓ Enterobacteriaceae, Pseudomonadaceae	Acetate, isobutyrate, butyrate, valerate ↑	Not measured (in vitro model)	Anti-inflammatory trend inferred from SCFA ↑ & pathogen suppression	Not performed	Tangeretin restored diversity and SCFAs, enriched beneficial taxa, and reduced opportunistic pathogens
HNP	Ren et al. [26]	American ginseng polysaccharide	Shannon ↑ (close to NC)	PCoA shifted toward NC	↑ Bacteroidetes; ↓ Firmicutes, Proteobacteria	Not reported	Colon structure repaired (↑ villus length, goblet cells)	Not reported	Villus and goblet cell morphology improved; edema reduced.	American ginseng polysaccharide alleviated dysbiosis and promoted mucosal repair via microbiota modulation.
HNP	Min, et al. [27]	Korean red ginseng polysaccharide	Diversity indices ↑	PCoA approached NC	↑ Firmicutes, ↓ Proteobacteria	Acetate and butyrate ↑	Lysozyme, claudin-1 ↑	Fecal IgA ↑	–	Red ginseng polysaccharide rebalanced microbiota, enhanced SCFA, and reinforced barrier and mucosal immune homeostasis.
HNP	Qi et al. [28]	Neutral ginseng polysaccharide	Shannon and Chao1 ↑	PCoA partially restored toward NC	↑ Lactobacillus; ↓ Bacteroides, Pseudomonas	Not measured	Histologic repair (villus structure)	Not reported	Villus atrophy and goblet cell loss reversed.	Neutral ginseng polysaccharide increased beneficial flora and reduced pathogens, restoring diversity and mucosal structure.
HNP	Pan et al. [29]	*Dioscorea* sulfated polysaccharide	Not reported (DGGE only)	UPGMA: closer to NC	↑ *Bacteroides thetaiotaomicron*; ↓ *Enterococcus*, *Acinetobacter*	Not reported	Not reported	Not reported	Not performed	Sulfated yam polysaccharide restored microbial balance and alleviated diarrhea via reduction in pathogens and enrichment of commensals.
HNP	Li S, et al. [30]	*Astragalus* polysaccharide	Shannon and Chao1 ↑	NMDS: closer to NC	↑ *Oscillospira*, *Dorea*; ↓ *Epulopiscium*, *Pseudomonas*	Propionate, butyrate, total SCFAs ↑	Colon architecture normalized	Inflammatory cell infiltration ↓	Normal mucosal architecture restored.	*Astragalus* polysaccharide modulated microbiota and selectively enhanced propionate and butyrate generation.
HNP	Xu et al. [31]	*Poria cocos* polysaccharide	Richness and diversity ↑	PCoA: closer to NC	↑ *Parabacteroides distasonis*, *Akkermansia muciniphila*; ↓ *Salmonella*, *Mucispirillum*	Predicted ↑ carbohydrate metabolism; SCFA receptors (GPR41/43) ↑	ZO-1, OC-1 ↑	FOXP3 mRNA ↑, NF-κB ↓	Mucosal damage reversed; villus and goblet cells normalized.	Poria polysaccharide restored microbial diversity and tight junctions, activated FOXP3 and SCFA receptor pathways, and suppressed NF-κB.
HNP	Lai et al. [32]	*Dictyophora* polysaccharide	Shannon and ACE ↑	PCoA/type analysis: DIPY shifted toward NC	↑ *Robinsoniella*, *Parasutterella*, *Blautia*; ↓ some *Muribaculaceae*	Acetate and total SCFAs ↑	Epithelial integrity and mucus layer restored	LPS, MCP-1, TNF-α, IL-6 ↓	Intact epithelium with restored goblet cells.	*Dictyophora* polysaccharide modulated microbiota, enhanced SCFA production, repaired barrier, and reduced systemic inflammation.
HNP	Bie et al. [33]	Sweet potato polysaccharide	Chao and Shannon ↑	PCoA: shifted toward NC	↓ Proteobacteria; ↑ Bacteroidetes, Muribaculaceae; ↓ *Escherichia coli*, *Klebsiella*	Acetate, propionate, butyrate, valerate, total SCFAs ↑	Ileal villus length and crypt depth restored	IL-10 ↑; IL-1β, IL-6, TNF-α ↓	Reduced inflammatory infiltration; improved ileal morphology.	Sweet potato polysaccharide reversed dysbiosis, enhanced SCFA production, and regulated IL-10–mediated anti-inflammatory pathways.
HNP	Zhang et al. [34]	Yam extract	DGGE: richness/evenness ↑	UPGMA/PCA: re-clustering toward NC	↑ *Bacteroides* spp., *Clostridium* spp.; ↓ *Enterobacter* spp.	Propionate, butyrate, valerate, total SCFAs ↑	Cecal index normalized (indirect barrier recovery)	Not assessed	Cecal enlargement reversed.	Yam extract acted as substrate for SCFA-producing bacteria, enhancing SCFA production and gut recovery.
HNP	Pan et al. [35]	Brown alga polysaccharide (*Nemacystus*)	Indices restored toward NC	PCoA: cluster closer to NC	↓ Proteobacteria; ↑ *Muribaculum*, *Lactobacillus*; ↓ *Enterobacter*, *Clostridioides*	SCFA-producing genera recovered (not quantified)	Occludin and SHIP ↑	IL-1β, IL-6, p-PI3K, p-NF-κB ↓	Smoother mucosa; reduced inflammatory infiltration.	Brown alga polysaccharide enhanced commensals, maintained tight junctions via SHIP–PI3K/NF-κB modulation, and suppressed inflammation.
HNP	Lai et al. [36]	*Poria cocos* water-insoluble polysaccharide	Simpson, ACE, Shannon ↑	PCoA: closer to NC	↑ *norank_f__Muribaculaceae*; ↓ *Staphylococcus*, *Acinetobacter*, *Escherichia–Shigella*	Acetate, butyrate ↑	Cecal mucosa continuity restored	Serum TNF-α, IL-6, IL-1β ↓	Almost normal architecture.	Water-insoluble Poria polysaccharide remodeled microbiota, increased SCFAs, reduced pro-inflammatory cytokines, and repaired barrier.
HNP	Lu et al. [37]	*Antrodia cinnamomea* intracellular polysaccharide	Shannon ↑, Simpson ↓ (diversity restored)	PCoA/NMDS: closer to NC	↑ Erysipelotrichaceae, Lachnospiraceae; ↓ *Enterococcus*	Not measured	Thymus and spleen index restored	Serum IL-6, TNF-α ↓	Colon structure improved.	*Antrodia* polysaccharide reduced pathogenic *Enterococcus*, enhanced butyrate-producing commensals, and improved immune-organ indices.
HNP	Cui et al. [38]	Sea cucumber (*Cereus sinensis*) polysaccharide	Shannon and Chao1 ↑	PCoA/NMDS/PLS-DA: closer to NC	Firmicutes/Bacteroidetes ratio normalized; ↑ *Phasecolarctobacterium*, *Bifidobacterium*	Acetate and total SCFA ↑; propionate and butyrate ↑	Cecal damage reduced; structure normalized	Serum IL-2, TNF-α ↓; IL-1β ↓ (high dose)	Cecal inflammation and edema reduced.	Sea cucumber polysaccharide enhanced SCFA-producing flora, improved SCFA output, and suppressed inflammatory cytokines.
HNP	Chen et al. [39]	Bamboo shoot polysaccharide	Shannon and Chao1 ↑	PCA: high-dose cluster closest to NC	F/B ratio normalized; ↑ *Lactobacillus*; ↓ *Escherichia–Shigella*	Acetate, propionate, butyrate, valerate ↑ (butyrate/valerate > inulin)	Thicker intestinal wall; reduced edema	No cytokine assay	Mucosal edema and inflammatory infiltration reversed.	Bamboo shoot polysaccharide was fermented in colon, increased SCFAs, promoted beneficial flora, and suppressed pathobionts.
HNP	Zeng. [40]	Blueberry leaf polyphenols	Diversity and richness restored	PCoA: clusters closer to NC	F/B ratio normalized; ↑ *Muribaculum*, *Lactobacillus*; ↓ *Clostridium*, *Enterococcus*	Acetate, propionate, butyrate, valerate, total SCFAs ↑	Occludin, claudin-1, ZO-1 ↑ (gene/protein)	Serum IL-1β, TNF-α, IL-6 ↓	Ileal necrosis and inflammatory infiltration repaired.	Blueberry leaf polyphenols repaired barrier, suppressed MAPK signaling, modulated microbiota, and promoted SCFA production.
HNP	Han et al. [41]	Cistanche extract/polysaccharide	Simpson and Shannon ↑	PCoA: clusters closer to NC	↓ Firmicutes; ↓ *Clostridium_sensu_stricto_1*; ↑ *Blautia*, Lachnospiraceae	Not measured	Not specified	Not specified	Colonic structure improved; inflammation reduced.	Cistanche effects were likely mediated by gut microbiota modulation and metabolite regulation.
HNP	Li et al. [42]	*Antrodia* polysaccharides (AEPS/AIPS)	Shannon and Simpson ↑	PCoA/NMDS: moved away from model cluster	AEPS: ↑ Lactobacillaceae; AIPS: ↑ Staphylococcaceae/*Staphylococcus*	Not reported	Not reported	Not reported	Not analyzed in detail.	*Antrodia* polysaccharides modulated gut microbiota; AEPS showed more favorable shift toward *Lactobacillus* dominance.
HNP	Ma et al. [43]	Fresh ginger extract	Diversity restored (after AAD-induced ↓)	PCoA/NMDS: ginger groups closer to NC	Proteobacteria ↓; *Escherichia–Shigella* ↓; *Bacteroides* ↑	Not reported	MUC2, ZO-1 ↑; goblet cells restored	MPO expression ↓; colonic inflammation ameliorated	Epithelial shedding and disorganized crypts reversed.	Fresh ginger suppressed *Escherichia–Shigella*, enhanced *Bacteroides*, and restored barrier integrity.
HNP	Zhang et al. [44]	Dried ginger extract	ACE/Shannon ↓ in model; ↑ with ginger	PCoA/cluster: ginger closer to NC	↓ *Bacillus*, *Lachnoclostridium*, *E. coli–Shigella*; ↑ *Lactobacillus*	Not measured	Not reported	Not reported	Epithelial shedding and crypt disruption improved.	Dried ginger restored diversity and beneficial *Lactobacillus* while reducing pathogenic *E. coli–Shigella*.
HNP	Li et al. [45]	American ginseng decoction	Richness ↔; Shannon/Simpson ↑	PCoA/NMDS: shifted toward NC	↓ Proteobacteria; ↑ Bacteroidetes/Firmicutes; ↓ *Pseudomonas*, *E. coli–Shigella*; ↑ *Bacteroides*	Not reported	Colon structure intact (H&E)	Inflammatory cell infiltration ↓	Loose glands and epithelial shedding improved.	American ginseng decoction improved diversity and composition and restored colon structure and metabolic pathways.
HNP-C	Qu et al. [46]	Fermented ginseng synbiotic	Simpson, Ace, Chao, Shannon ↑ (restored)	PCA: fermented ginseng cluster closest to NC	↑ *Lactobacillus*, *Candidatus Stoquefichus*; ↓ *Bacteroides*, *Clostridioides*	Not measured	Crypt architecture normalized; infiltration ↓	IL-1β, IL-6, TNF-α ↓; IL-10 ↑	Colon architecture restored.	Fermentation increased β-glucosidase activity, boosted bioactive ginsenosides, normalized microbiota and cytokine balance.
HNP-C	Zhong et al. [47]	*Astragalus* polysaccharide + *L. plantarum*	α-diversity ↑ (combination > single > model)	PCoA: combination closest to NC	↑ *Lactobacillus*, *Allobaculum*, *Bifidobacterium*; ↓ *Bacteroides*, *Blautia*	Not measured	Occludin, claudin-1, ZO-1, MUC-2 ↑; goblet cells ↑; DAO, D-LA, LPS ↓	sIgA, IgG ↑; IL-17A, IL-4, TGF-β1 ↓	Goblet cells and mucus area increased; edema ↓	Astragalus synbiotic enhanced tight junctions and mucus, improved immunoglobulin profile, and promoted epithelial repair via Smad7/p-Smad3 modulation.
HNP-C	Tang et al. [48]	Poria polysaccharide + probiotics	Chao and Shannon ↑ (PWP > PP > WP)	PCoA/NMDS: PWP cluster closest to NC	↑ Firmicutes, Bacteroidetes (Muribaculaceae); ↓ Proteobacteria (*Sutterella*)	Not measured	Mucin ↑ (histology)	IgM, IgG normalized; IgA ↑; macrophages/lymphocytes ↑	Mucin ↑; edema ↓; improved villus morphology.	Poria-based synbiotic was associated with immune modulation and microbiota homeostasis, with more pronounced changes observed under the tested conditions.
HNP-C	Guo et al. [49]	*B. adolescentis* + β-glucan synbiotic	Shannon and Fisher ↑ (combination > single)	PCoA: combination cluster closest to NC	↑ *B. uniformis*; ↓ *Enterococcus*, *Escherichia–Shigella*	Acetate, propionate, isobutyrate ↑	Occludin, ZO-1, Mucin-2/3 ↑; D-LA, LPS ↓	IL-6, IL-17 ↓	Mucosal structure improved; epithelium intact.	Synbiotic increased SCFAs, repaired barrier, and modulated immunity via enrichment of *B. uniformis*.
HNP-C	Li et al. [50]	Maifan stone + *L. rhamnosus* GG	Not reported	Not applicable (qPCR only)	↑ *Bacteroides*, *Lactobacillus*; ↓ *Enterococcus*, *E. coli*	Not measured	Colon epithelium arrangement restored; edema ↓	TLR4, NF-κB ↓ in colon	Edema reduced; epithelial structure normalized.	Maifan stone carrier enhanced LGG tolerance, improved microbiota, and down-regulated TLR4/NF-κB signaling.
HNP-C	Shen et al. [51]	Chitosan oligosaccharide + probiotics	Shannon ↑; Chao1 trend toward recovery	PCoA: synbiotic group closer to baseline (day 0)	↑ Firmicutes, Acidobacteriota; ↓ Desulfobacterota; shifts in *Megamonas*, Lachnospiraceae, Muribaculaceae	Not measured	ZO-1, occludin, claudin-1 ↑; villus height/crypt depth ↑	TNF-α, IL-1β ↓; IgA, IgG ↑	Edema ↓; villus height and crypt depth improved.	Chitosan oligosaccharide acted as prebiotic carrier; synbiotic restored tight junctions and reduced inflammation in a canine model.

HNP = Herbal/Natural Product; HNP-C = Herbal/Natural Product Combination; ↑ = increase; ↓ = decrease.

**Table 3:** Comparative Effects Between HNP Monotherapy and HNP-C Combination Therapies.

Outcome	HNP Monotherapy (*n* = 21)	HNP-C Combination (*n* = 6)	Comparative Interpretation
α-Diversity	Reported in 20/21 studies; 1 study used DGGE only (no α-diversity indices). All studies reported ↑ Shannon/Chao1/ACE.	Reported in 5/6 studies; one study [45,50] did not assess α-diversity. All reporting studies showed increases.	Both strong; HNP-C slightly more rapid and consistent in studies with direct comparators.
β-Diversity	Reported in 18/21 studies (PCoA/NMDS). All 18 showed a shift toward Control; 3 did not report β-diversity.	Reported in 5/6 studies; Li et al. [45,50] used qPCR only. All reporting studies showed convergence toward NC.	HNP-C tends to show closer clustering toward the Control group in studies reporting β-diversity.
Key taxa shifts	Reported in 21/21 studies. Common findings: ↑ *Lactobacillus*, *Bifidobacterium,* Muribaculaceae; ↓ *Escherichia–Shigella.*	Reported in 6/6 studies. Persistence of administered probiotic strains.	Similar direction overall; HNP-C yields more stable probiotic colonization.
SCFAs	Reported in 17/21 studies. All reported ↑ acetate/propionate/butyrate.	Quantitatively reported in 1/6 studies [49]. Other 5 did not measure SCFAs directly; some reported ↑ SCFA-producing taxa.	HNP monotherapy provides stronger direct evidence for SCFA production (17/21 studies). HNP-C efficacy is currently limited by reporting frequency (1/6 studies) but supported indirectly.
Barrier markers (ZO-1, Occludin, Claudin-1, MUC2, SIgA)	Reported in 16/21 studies; improved TJ proteins and mucosal integrity.	Reported in 4/6 studies; increased TJ proteins and reduced leakage markers (D-LA, LPS).	HNP-C showed improvements across multiple barrier-related markers when reported.
Pro-inflammatory cytokines (TNF-α, IL-1β, IL-6)	Reported in 15/21 studies; consistent decreases.	Reported in 6/6 studies; also ↓ IL-17A and ↑ IL-10.	HNP-C showed modulation across multiple cytokine markers.
Immune markers (IgA, IgG, IgM, FOXP3)	Reported in 11/21 studies; some ↑ IgA and ↑ FOXP3.	Reported in 5/6 studies; all showed ↑ IgA/IgG/IgM.	HNP-C showed consistent improvements in immune-related outcomes.
Diarrhea-related phenotypic outcomes	Reported in 18/21 studies; improved diarrhea severity and weight recovery.	Reported in 6/6 studies; all showed. phenotypic improvement	Both effective; HNP-C shows more complete and consistent reporting.
Histopathology	Reported in 17/21 studies; mucosal repair, ↑ goblet cells, ↓ edema.	Reported in 6/6 studies; villus height ↑, crypt integrity restored.	Comparable effects; HNP-C shows uniformly positive histology.
Overall consistency and strength	Mechanisms vary by herb/extract; reporting frequency variable.	Highly coherent across barrier, immunity, taxa, and histology.	HNP-C consistently showed recovery across multiple domains. The two direct comparator studies suggest potentially additive benefits across microbial, barrier, and immune endpoints.

↑ = increase; ↓ = decrease.

### 3.4. Functional Recovery: SCFA Metabolism and Barrier Integrity

SCFA metabolism and epithelial barrier function were evaluated with varying depth across studies (Table 3). Among HNP interventions, 17 of the 21 studies quantified SCFAs [25,27,29,31,33,35,37,38,39,41,42,43,44,45] and consistently reported increases in total SCFAs or specific metabolites such as acetate, propionate, and butyrate. These biochemical improvements generally corresponded with the restoration of SCFA-producing taxa, including Lachnospiraceae and Ruminococcaceae. In contrast, only one of the six HNP-C studies directly quantified SCFAs [49]. The remaining studies inferred metabolic improvement through increased abundance of SCFA-producing genera without direct metabolite measurements [46,47,48,50,51]. Consequently, current evidence for SCFA enhancement is substantially stronger for HNP monotherapy than for HNP-C combinations. In contrast, SCFA-related findings in HNP-C studies were largely inferred from shifts in SCFA-producing taxa rather than direct metabolite quantification.

Barrier-related markers were more consistently assessed. Eighteen HNP studies [26,27,28,31,32,33,35,36,37,38,39,40,41,42,43,44,45] and four HNP-C studies [47,48,49,50,51] demonstrated improvements in tight junction proteins (ZO-1, occludin, claudin-1), mucin production (MUC2), permeability-related biomarkers (DAO, D-LA, LPS), and mucosal histology. Notably, only two HNP-C studies incorporated comparator groups that included herbal-only, probiotic-only, and combination arms [47,48], and both more pronounced recovery of epithelial structure and permeability with combination therapy. The remaining HNP-C studies [46,49,50,51] showed similar benefits but lacked designs that allow evaluation of additive effects. Overall, both HNP and HNP-C interventions were associated with functional recovery from antibiotic-induced intestinal injury; however, mechanistic evidence supporting additive benefits in HNP-C interventions remains limited to two well-controlled studies.

### 3.5. Immune and Inflammatory Responses

Fifteen HNP studies assessed immune or inflammatory markers [25,27,28,29,32,33,34,36,37,38,39,41,42,44,45] and generally reported reductions in pro-inflammatory cytokines (TNF-α, IL-1β, IL-6), with several studies also observing increases in IL-10 or SIgA, suggesting partial immune normalization concurrent with microbiota recovery. All six HNP-C studies evaluated immune outcomes [46,47,48,49,50,51] and consistently showed reduced inflammatory cytokine expression and/or enhanced immunoglobulin levels. Among these, two studies employed direct comparator designs [47,48] and reported more pronounced cytokine reduction and humoral immune changes in the combination groups. The remaining studies [46,49,50,51] showed similar trends but lacked the methodological elements necessary to confirm synergistic effects.

Taken together, both intervention categories appeared to mitigated antibiotic-induced inflammation. Although HNP-C regimens may provide potentially complementary immunomodulatory effects, current evidence supporting this hypothesis is restricted to two rigorously controlled comparator studies.

### 3.6. Histopathological Recovery

Histological evaluation was conducted in 23 studies, including 17 HNP studies [26,28,31,33,35,36,37,38,39,40,42,43,44,45] and all six HNP-C studies [46,47,48,49,50,51]. Both intervention types improved villus and crypt architecture, reduced mucosal edema, restored goblet cell density, and decreased inflammatory cell infiltration. In most studies, histopathological outcomes were evaluated qualitatively based on morphological criteria, as standardized scoring systems were not consistently applied across the included studies. Among HNP-C trials, two studies with direct comparator arms [47,48] showed more pronounced mucosal recovery in the combination groups than in the respective monotherapy groups. The remaining HNP-C studies [46,49,50,51] reported similar improvements but lacked designs that permitted assessment of synergistic effects. Overall, histological repair was consistently observed across all included studies and generally paralleled improvements in microbiota composition, metabolic activity, and immune function.

### 3.7. Diarrhea-Related Phenotypic Outcomes

Clinical symptom assessments were reported in 18 HNP studies [25,26,27,28,29,31,32,33,35,36,37,38,39,40,41,42,43,44,45] and all HNP-C studies [46,47,48,49,50,51]. Improvements included reduced diarrhea severity, enhanced stool consistency, and accelerated weight recovery, changes that likely reflect ongoing microbial and mucosal restoration. Only two HNP-C studies [47,48] incorporated suitable comparator groups, and both showed faster or more pronounced symptomatic recovery in the combination groups than in monotherapy. Other HNP-C studies showed comparable diarrhea-related phenotypic benefits but lacked design features necessary to evaluate synergy. Overall, phenotypic improvements were consistently observed across both intervention categories, with evidence of additive benefit from combined HNP and probiotic administration emerging only in studies with appropriate comparative frameworks.

### 3.8. Comparative Effects Between HNP and HNP-C Using Overlapping Herbal Sources

Two key herbal ingredients, Poria (Poria/*Wolfiporia cocos*) and Astragalus (*Astragalus* spp.), were included in both HNP monotherapy and HNP-C interventions. Despite differences in extraction and formulation, these overlapping components provided the only basis for evaluating whether the addition of probiotics yields benefits beyond monotherapy. Notably, most HNP monotherapy studies used two-group designs (monotherapy vs. control), whereas the HNP-C studies employing these herbs used three-group designs (HNP monotherapy vs. probiotic monotherapy vs. combination therapy), allowing clearer assessment of potential synergistic effects.

#### 3.8.1. Poria (*Wolfiporia cocos*) Group (Table 4)

A series of Poria-based HNP monotherapy studies [32,36] reported consistent improvements in epithelial barrier integrity, including restored ZO-1 and occludin expression, reduced NF-κB activation, and recovery of mucosal architecture. In contrast, the Poria-containing HNP-C study [48] employed a three-arm design that isolated the effects of each therapeutic component. The combination of Poria polysaccharides with probiotics produced more comprehensive biological benefits than either component alone, including enhanced mucus secretion and goblet cell density, greater recovery of tight-junction structure, increased humoral immune responses (IgA/IgG/IgM), and a more sustained re-establishment of gut microbial community structure over time. These findings suggest that although Poria monotherapy consistently supports epithelial repair, co-administration with probiotics may provide complementary improvements across microbial, barrier, and immune domains, indicating potential additive therapeutic advantages under the tested conditions.

**Table 4:** Poria Group (*Wolfiporia cocos*).

Item	HNP (PCP, PCY)	HNP-C (WP + Probiotics)
Barrier	↑ ZO-1, Occludin; epithelial repair	↑ mucin, ↑ goblet cells; ↓ leakage markers
Immune	↓ NF-κB; GPR41/43 ↑	↑ IgA/IgG/IgM; ↑ macrophage/lymphocyte activity
Microbiota	Restored Firmicutes/Bacteroidetes; ↓ pathogens	Relatively stable community structure; sustained shifts
Histology	Edema ↓; villus repair	More pronounced epithelial regeneration
Mechanism	SCFA receptor (GPR41/43) activation	Combined barrier and immune modulation

This table presents descriptive comparisons based on individual studies and is intended for hypothesis-generating purposes. ↑ = increase; ↓ = decrease.

#### 3.8.2. Astragalus (*Astragalus* spp.) Group (Table 5)

The efficacy of Astragalus-based HNP monotherapy [29] was evaluated using standard comparisons between the control group, AAD model group, and herbal monotherapy group. In this study, Astragalus polysaccharide administration supported recovery of microbial diversity, selectively enriched SCFA-producing taxa, promoted villus restoration, and increased levels of propionate and butyrate. In contrast, the Astragalus-containing HNP-C intervention [47] used a three-group design comparing herbal therapy, probiotic therapy, and combined treatment, enabling clearer differentiation of each component’s effects. In this design, the synbiotic combination produced more coordinated improvements than either monotherapy, including greater upregulation of tight-junction and mucus-related proteins (Occludin, Claudin-1, ZO-1, MUC2), enhanced humoral immune responses (IgA/IgM/IgG), and larger reductions in permeability-related markers (DAO, D-lactic acid, LPS). Additionally, modulation of Smad7/p-Smad3 signaling offered further mechanistic support for epithelial regenerative activity. Overall, while Astragalus monotherapy primarily improved microbial composition and SCFA-associated recovery, combining Astragalus with probiotics resulted in more comprehensive and coordinated improvements across microbial, barrier, and immune domains, suggesting a potential additive advantage under the tested conditions.

**Table 5:** Astragalus Group.

Item	HNP (WAP)	HNP-C (APS + *L. plantarum*)
SCFAs	↑ propionate, ↑ butyrate	Not directly measured
Barrier	Villus restoration	↑ Occludin/Claudin-1/ZO-1/MUC-2; ↓ DAO/D-LA/LPS
Immune	↓ inflammation	↑ IgA/IgM/IgG
Mechanism	Microbiota + SCFA effects	↑ Smad7; ↓ p-Smad3; pathway-level modulation

This table presents descriptive comparisons based on individual studies and is intended for hypothesis-generating purposes. ↑ = increase; ↓ = decrease.

#### 3.8.3. Integrated Summary

Across both Poria and Astragalus categories, herbal monotherapies improved dysbiosis, barrier integrity, and inflammatory responses, while combination therapies produced more comprehensive and coordinated recovery patterns across microbial, immunological, and structural outcomes. These patterns were most evident in the three-arm designs used in the combination studies, which showed that dual-component interventions were associated with more extensive improvements than herbal-only or probiotic-only treatments under the tested conditions. Although the number of overlapping interventions is limited, the available evidence suggests that combining herbal materials with probiotic strains may support broader and more stable recovery profiles in antibiotic-associated diarrhea.

### 3.9. Risk of Bias and Quality of Reporting

**Figure 2:**
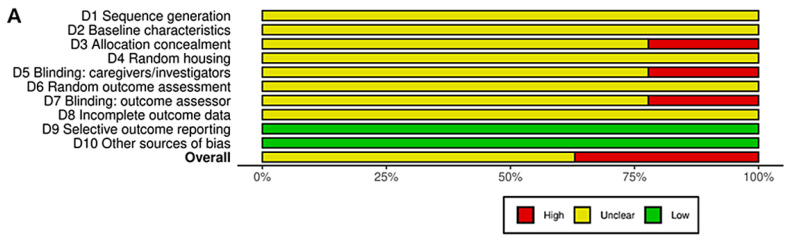

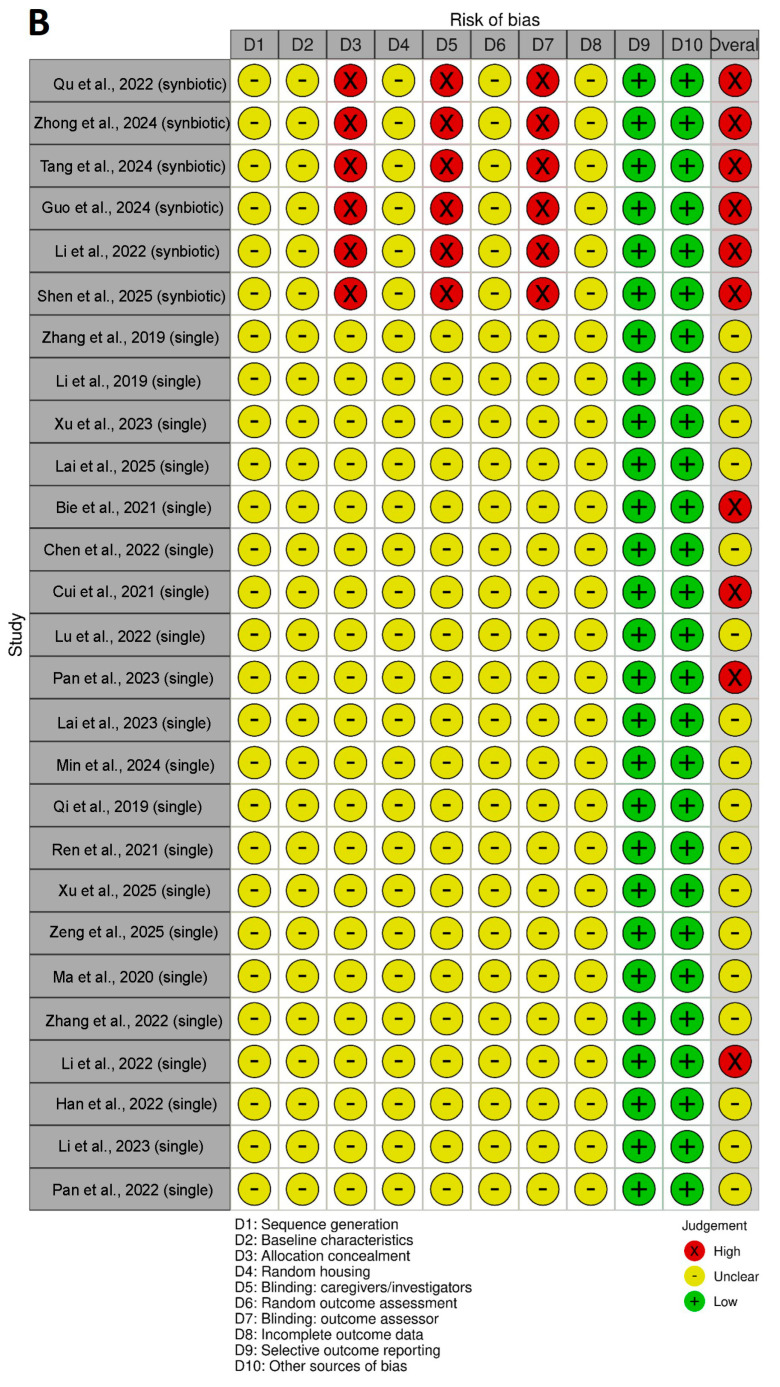
SYRCLE risk-of-bias assessment of included animal studies [25,26,27,28,29,30,31,32,33,34,35,36,37,38,39,40,41,42,43,44,45,46,47,48,49,50,51]. (**A**) Summary of risk-of-bias ratings across all SYRCLE domains, presented as the percentage of studies classified as low, high, or unclear risk. (**B**) Risk-of-bias evaluation for each included study, with individual SYRCLE items scored as yes (low risk), no (high risk), or unclear (−).

The methodological quality of the 27 included animal studies was assessed using the SYRCLE risk-of-bias tool (Figure 2). Reporting of key study design safeguards was generally insufficient across both HNP (*n* = 21) and HNP-C (*n* = 6) interventions. Although many studies stated that animals were randomly assigned to groups, none described the method of sequence generation, resulting in unclear risk for selection bias. Allocation concealment was also not reported in any study and was therefore judged as high or unclear risk. Blinding of caregivers, investigators, or outcome assessors was rarely described, contributing to predominantly unclear ratings for performance and detection bias. In contrast, selective reporting and outcome completeness were generally adequate, and most studies were rated as low risk in these domains. Overall, the frequent “unclear” assessments stemmed from insufficient methodological reporting rather than evidence of systematic flaws. Future preclinical studies should clearly detail randomization methods, allocation concealment, and blinding procedures to strengthen the robustness and interpretability of evidence.

## 4. Discussion

Understanding the therapeutic landscape of AAD requires consideration not only of the immediate loss of microbial diversity but also of the broader functional disruptions caused by antibiotic exposure [25,26,27,28,29]. Antibiotics suppress SCFA metabolism, compromise epithelial tight junction integrity, reduce mucus secretion, and disturb mucosal immune regulation, alterations that collectively shape the trajectory of post-antibiotic recovery [31,32,33,34,35,41,42,43,44,45]. These multidimensional disturbances also help explain why conventional strategies such as probiotics often yield incomplete benefits, particularly when microbial colonization remains hindered under antibiotic pressure [46,47,48,49]. 

Within this context, HNPs have emerged as promising multi-target interventions. Many HNP compounds influence microbial pathways such as enriching beneficial commensals, suppressing opportunistic bacteria, and partially normalizing microbial community structure while also modulating host processes related to epithelial repair, barrier reinforcement, and immune regulation [32,36,37,38,41,42,43,44,45]. Earlier preclinical studies showed these benefits separately, such as histological improvement or reduced inflammation, but were unable to link these outcomes to microbiota restoration due to the lack of sequencing data [17,18,19].

Accordingly, HNP–C strategies have gained traction as an integrated therapeutic approach. This strategy aligns with principles of rational synbiotic design, aiming to address the metabolic and colonization limitations of single-agent therapy by using HNPs as fermentable substrates that promote probiotic colonization and functional output [52,53].

In this review, several integrated patterns emerged. Most HNP monotherapy studies that included microbial endpoints demonstrated restoration of diversity and community structure alongside improvements in barrier and immune markers [25,26,27,28,29,30,31,32,33,34,35,36,37,38,39,40,41,42,43,44,45], suggesting that ecological and host-functional recovery progress concurrently [3,4,54,55]. Seventeen of the 21 monotherapy studies measured SCFAs and reported increases in acetate, propionate, or butyrate [25,27,29,31,33,35,37,38,39,41,42,43,44,45]. Although the analytical platforms varied and quantitative synthesis was not feasible, the consistency of these findings supports the interpretation that HNP-driven metabolic recovery is a downstream outcome of microbiota restoration and is essential for epithelial barrier function [56,57].

### 4.1. Effects of HNP Monotherapy

HNP monotherapies demonstrated coherent recovery across ecological and host-functional domains. Twenty of the 21 studies reported increases in α-diversity [25,26,27,28,29,30,31,32,33,34,35,36,37,38,39,40,41,42,43,44,45], and 18 studies showed β-diversity shifts toward healthy control clustering [25,26,27,28,29,31,32,33,35,36,37,38,39,40,41,42,43,44,45]. Beneficial commensals, including *Lactobacillus*, *Bifidobacterium*, Muribaculaceae, Lachnospiraceae, and Ruminococcaceae expanded, whereas Proteobacteria-dominant taxa, such as *Escherichia–Shigella*, diminished, indicating that microbial restoration and mucosal recovery likely progress in parallel [3,4,54,55]. SCFA profiles also improved consistently across studies that quantified SCFAs [25,27,29,31,33,35,37,38,39,41,42,43,44,45], aligning with the regrowth of SCFA-producing taxa. These ecological gains coincided with increased tight-junction expression [26,27,28,31,32,33,35,36,37,38,39,40,41,42,43,44,45], reduced permeability markers [26,27,28,31,32,33,35,36,37,38,39,40,41,42,43,44,45], and decreased pro-inflammatory cytokines [25,27,28,29,32,33,34,36,37,38,39,41,42,44,45]. Collectively, these patterns suggest that HNPs exert primary microbiota-mediated effects, with secondary improvements emerging through enhanced barrier integrity and immune regulation [56,57].

### 4.2. Effects of HNP–Probiotic Combinations (HNP-C)

Although fewer in number, HNP-C studies demonstrated more comprehensive and coordinated improvements across microbial, epithelial, and immune endpoints [46,47,48,49,50,51]. This integrated recovery aligns with principles of rational synbiotic design, wherein functional complementarity between the herbal substrate and probiotic strain facilitates coordinated biological responses that may exceed simple additive effects [52,53]. All six studies reported improved α-diversity and β-diversity trajectories moving toward healthy control clustering [46,47,48,49,50,51]. Combination therapies consistently increased tight-junction markers, reduced permeability indices, and produced broader immunologic modulation, including elevations in IgA, IgM, and IgG [46,47,48,49,50,51]. The two trials using three-arm designs directly comparing HNP-only, probiotic-only, and HNP-C groups offered particularly valuable insights [47,48]. In both studies, the combined intervention showed greater improvements across ecological, barrier, and immunologic outcomes compared with monotherapy, suggesting potential additive or context-dependent synergistic interactions. However, because only one study quantified SCFAs directly [49] and comparator designs remain limited, existing evidence is insufficient to conclude generalizable superiority of combination therapies.

### 4.3. Probiotics vs. HNP vs. HNP–C: Comparative Mechanistic Insights

The probiotic comparator groups primarily used *Lactobacillus* and *Bifidobacterium* species or representative strains such as *Lactiplantibacillus plantarum* ELF051 and *Bifidobacterium adolescentis*. Across the four studies that included probiotic-only arms [47,48,49,50], these strains consistently improved selected immune parameters, such as increased SIgA or reduced IL-6 and TNF-αand produced modest enhancement of mucin expression. However, consistent with their metabolic profiles and the strain-dependent nature of SCFA production, probiotic-only interventions demonstrated limited restoration of bulk SCFA concentrations, α-diversity indices, and major commensal families disrupted by antibiotics (e.g., *Lachnospiraceae*, *Ruminococcaceae*, *Muribaculaceae*) [47,48,49,50,58].

In contrast, HNP monotherapies in the same comparator studies promoted the recovery of these commensal families by supplying selectively utilizable polysaccharides and complex carbohydrates that serve as fermentation substrates [59]. These microbiota-dependent effects were often accompanied by direct host-targeted actions, including reinforcement of tight-junction structure and modulation of NF-κB–associated inflammatory pathways, which help explain why HNP monotherapy frequently matched or exceeded probiotic-only interventions in restoring epithelial integrity and metabolic function.

The complementary strengths of both components were most evident in the three-arm comparator designs. Combination groups showed closer β-diversity clustering toward healthy controls, greater upregulation of epithelial proteins (ZO-1, Occludin, MUC2), and more extensive immunological modulation than either monotherapy. These outcomes are consistent with a mechanistic model in which HNPs provide structural and metabolic scaffolding that supports microbial viability, while probiotics accelerate recolonization and mucosal immune coordination, resulting in more coordinated ecosystem recovery [52,53,60].

Taken together, HNP-C therapies appear to leverage potentially synergistic or additive interactions, pairing the ecological rebuilding capacity of HNPs with the targeted recolonization and immune-signaling strengths of probiotics to achieve more comprehensive and coordinated recovery profiles than either component alone under comparator-controlled conditions.

### 4.4. Comparative Insights from Overlapping Herbal Sources

Parallel evaluation of herbs used in both monotherapy and HNP-C formulations, such as Poria and Astragalus, offered additional mechanistic insight. Poria monotherapy improved microbial diversity, strengthened tight-junction structure, and reduced NF-κB activation, whereas the corresponding combination enhanced mucin secretion, goblet cell density, and immunoglobulin levels. Similarly, Astragalus monotherapy increased propionate and butyrate levels and supported villus restoration, while the combination further reinforced tight-junction expression, reduced permeability, and modulated Smad7/p-Smad3 signaling. Although the number of these paired comparisons was limited and based on study-level observations rather than direct head-to-head designs, these findings suggest that overlapping HNP substrates and probiotic components may exert complementary effects on microbial, epithelial, and immune-related outcomes under the tested conditions.

### 4.5. Future Directions

Based on the patterns identified, future research should address several strategic priorities across both preclinical and clinical domains. In the preclinical setting, methodological rigor is essential, including the adoption of three-arm comparator designs (HNP vs. probiotic vs. HNP-C) to robustly evaluate additive and interaction effects. Methodological standardization is also critical and should include harmonized antibiotic-induction protocols, aligned microbiome sampling time points, consistent α-/β-diversity metrics, and a comprehensive assessment of SCFAs, tight-junction markers, and immune endpoints. Beyond standardization, greater mechanistic depth will require the integration of multi-omics approaches (e.g., metabolomics, transcriptomics) and the identification of strain–substrate –matched synbiotic pairs based on fermentation compatibility. For clinical translation, priorities include incorporating microbiome endpoints (diversity, SCFAs, barrier biomarkers) and implementing time-aligned sampling during and after antibiotic therapy. These foundational data will support rational synbiotic trial design using mechanistically complementary herbal–probiotic combinations in Phase II RCTs comparing HNP, standard care, and synbiotic formulations.

### 4.6. Overall Synthesis and Conclusions

This review provides an integrated evaluation of microbiota restoration and host-functional outcomes, addressing the multidimensional nature of recovery in antibiotic-associated diarrhea. By synthesizing ecological indices, SCFA metabolism, barrier integrity, immune responses, and histopathological changes, these findings offer a more comprehensive mechanistic perspective than previous work that assessed only isolated endpoints.

However, certain methodological limitations restrict translational certainty. Heterogeneity across antibiotic protocols, animal strains, intervention durations, and microbiome analytic methods, together with incomplete reporting of randomization, allocation concealment, and blinding, resulted in predominantly unclear or high risks of bias. Accordingly, comparative and mechanistic interpretations—particularly regarding the relative advantages of HNP–C strategies and inferred signaling pathways—should be regarded as hypothesis-generating rather than confirmatory. Furthermore, in the absence of functional validation (e.g., pathway inhibition), observed changes in signaling markers should be regarded as associative rather than causal and interpreted with caution. Finally, only one HNP–C study directly quantified SCFAs, limiting conclusions regarding potential metabolic benefits, which remain largely inferred from microbiota compositional shifts rather than from directly quantified outcomes.

## Figures and Tables

**Table 1:** Study characteristics of the included HNP and HNP-C interventions.

Study ID	Intervention Type	Intervention (Source Herb—Fraction/Extract + Dose/Duration)	Animal Model	AAD Induction (Agent, Duration)	Groups (*n*)	Comparator Arms	Treatment Duration	Total Period	Microbiome Method
Xu et al. [25]	HNP	*Citrus reticulata*—Tangeretin flavonoid (250 ppm, 10 days; co-administered with antibiotics)	C57BL/6 J mice	Ampicillin + Neomycin, 10 days	*n* = 8 × 2	AB-only vs. HNP-only	10 days	10 days	16S rRNA (V3–V4), in vitro fermentation
Ren et al. [26]	HNP	*Panax quinquefolius*—Water-soluble polysaccharide (100 mg/kg, 7 days)	Wistar rats	Clindamycin phosphate, 5 days	*n* = 6 × 4	NC/Model/NR/HNP-only	7 days	12 days	16S rRNA sequencing
Min, et al. [27]	HNP	*Panax ginseng*—Polysaccharide fraction (100/300 mg/kg, 12 days)	BALB/c mice	Lincomycin, 9 days	*n* = 7 × 4	NC/Model/HNP-low/high	12 days	21 days	16S rRNA
Qi et al. [28]	HNP	*Panax ginseng*—Neutral polysaccharide (dose NR, 7 days)	BALB/c mice	Lincomycin hydrochloride, 3 days	*n* = 10 × 4	NC/Model/NR/HNP-only	7 days	10 days	16S rRNA (V3–V4)
Pan et al. [29]	HNP	*Dioscorea opposita*—Sulfated polysaccharide (30 mg/kg, 14 days; antibiotics days 1–7)	Mice	Mixed antibiotics, 7 days	*n* = 8 × 3	NC/Model/HNP-only	14 days	14 days	16S rRNA DGGE
Li S, et al. [30]	HNP	*Astragalus membranaceus*—Water-soluble polysaccharide (100 mg/kg, 7 days)	Wistar rats	Lincomycin hydrochloride, 4 days	*n* = 6 × 4	NC/Model/NR/HNP-only	7 days	11 days	16S rRNA (V3–V4)
Xu et al. [31]	HNP	*Poria cocos*—Water-soluble polysaccharide (250 mg/kg/day, 7 days)	Mice	Broad-spectrum antibiotics, 7 days	*n* = 6 × 4	NC/AB-only/HNP-only/Positive Control	7 days	14 days	16S rDNA
Lai et al. [32]	HNP	*Dictyophora indusiata*—Water-insoluble polysaccharide (300 mg/kg, 7 days)	C57BL/6 J mice	Lincomycin hydrochloride, 3 days	*n* = 7 × 3	NC/Model (NR-like)/HNP-only	7 days	10 days	16S rRNA + GC–MS
Bie et al. [33]	HNP	*Ipomoea batatas*—Polysaccharide fraction (0.1–0.4 g/kg, 14 days)	BALB/c mice	Lincomycin hydrochloride, 3 days	*n* = 10 × 5	NC/Model/HNP (Low/Mid/High)	14 days	17 days	16S rRNA
Zhang et al. [34]	HNP	*Dioscorea opposita*—Water extract (dose NR, 10 days)	BALB/c mice	Ampicillin, 5 days	*n* = 10 × 5	NC/Model/HNP (Low/Mid/High)	10 days	15 days	PCR-DGGE
Pan et al. [35]	HNP	*Nemacystus decipiens*—Polysaccharide fraction (30 mg/kg/day, 14 days)	C57BL/6 mice	Four-antibiotic mix, 7 days	*n* = 8 × 3	NC/Model/HNP-only	14 days	21 days	16S rRNA
Lai et al. [36]	HNP	*Poria cocos*—Water-insoluble polysaccharide (300 mg/kg/day, 7 days)	C57BL/6 mice	Lincomycin hydrochloride, 3 days	*n* = 7 × 4	NC/Model/HNP-only/NR	7 days	10 days	16S rRNA
Lu et al. [37]	HNP	*Antrodia cinnamomea*—Intracellular polysaccharide (8 days)	ICR mice	Lincomycin hydrochloride, 3 days	*n* = 7 × 6	NC/Model/HNP (L/M/H)/FOS (PC)	8 days	11 days	16S rRNA
Cui et al. [38]	HNP	*Cereus sinensis*—Polysaccharide (75–300 mg/kg, 9 days)	C57BL/6 mice	Lincomycin hydrochloride, 3 days	*n* = 3 × 6	NC/Model/NR/HNP (L/M/H)	9 days	12 days	16S rRNA + GC–MS
Chen et al. [39]	HNP	*Leleba oldhami*—Bamboo-shoot polysaccharide (100–400 mg/kg, 15 days)	Kunming mice	Lincomycin hydrochloride, 3 days	*n* = 6 × 6	NC/Model/Inulin (PC)/HNP (L/M/H)	15 days	18 days	16S rRNA + SCFA detection
Zeng. [40]	HNP	*Vaccinium corymbosum*—Leaf polyphenol extract (100–300 mg/kg, 14 days)	BALB/c mice	Lincomycin hydrochloride, 3 days	*n* = 10 × 6	NC/Model/HNP (L/M/H)/GOS (PC)	14 days	17 days	16S rRNA + GC–MS
Han et al. [41]	HNP	*Cistanche deserticola*—Extract/polysaccharide/echinacoside (7 days)	BALB/c mice	Lincomycin hydrochloride, 7 days	*n* = 8 × 6	NC/Model/Inulin (PC)/HNP fractions	7 days	14 days	16S rDNA
Li et al. [42]	HNP	*Antrodia cinnamomea*—Polysaccharide fractions (0.03 g/kg, 3 days)	ICR mice	Lincomycin hydrochloride, 3 days	*n* = 6 × 5	NC/Model/HNP fractions/FOS (PC)	3 days	6 days	16S rDNA
Ma et al. [43]	HNP	*Zingiber officinale*—Fresh ginger extract (1.2 or 4.8 g/kg/day, 7 days)	SD rats	Clindamycin + Ampicillin + Streptomycin, 7 days	*n* = 10 × 4	NC/Model/HNP-low/high	7 days	21 days	16S rRNA
Zhang et al. [44]	HNP	*Zingiberis rhizoma*—Dried ginger extract (200–400 mg/kg/day, 14 days)	SD rats	Same three-antibiotic mix, 7 days	*n* = 10 × 5	NC/Model/HNP-low/high/PC	14 days	21 days	16S rRNA
Li et al. [45]	HNP	*Panax quinquefolium*—Decoction extract (1–2 g/kg/day, 7 days)	SD rats	Three-antibiotic mix, 7 days	*n* = 10 × 4	NC/Model/HNP-low/high	7 days	21 days	16S rRNA
Qu et al. [46]	HNP-C	*Panax ginseng* (fermented with *Limosilactobacillus fermentum*)—Fermented ginseng mixture	SD rats	Cephalexin + Clindamycin + Streptomycin, 7 days	*n* = 8 × 5	NC/Model/HNP-C/Probiotic-only/Mixed Combo	5 days	12 days	16S rRNA
Zhong et al. [47]	HNP-C	*Astragalus membranaceus* polysaccharide + *Lactiplantibacillus plantarum* ELF051	C57BL/6 mice	Amoxicillin + Clindamycin + Streptomycin, 14 days	*n* = 10 × 5	NC/Model/HNP-only/PRO-only/HNP-C	14 days	28 days	16S rRNA
Tang et al. [48]	HNP-C	*Wolfiporia cocos* polysaccharide + probiotic mixture (*Lactobacillus*, *Bifidobacterium*, *Enterococcus*)	Kunming mice	Ampicillin sodium, 5 days	*n* = 10 × 5	NC/Model/HNP-only/PRO-only/HNP-C	14 days	19 days	16S rRNA + CFU
Guo et al. [49]	HNP-C	*Bifidobacterium adolescentis* + yeast β-glucan	BALB/c mice	Lincomycin hydrochloride, 3 days	*n* = 10 × 6	NC/Model/HNP-only/PRO-only/HNP-C/NR	7 days	10 days	16S rRNA + metabolomics
Li et al. [50]	HNP-C	Maifan stone + *Lactobacillus rhamnosus* GG	SD rats	Clindamycin + Cephalexin + Streptomycin, 5 days	*n* = 10 × 6	NC/Model/NR/PRO-only/HNP-only/HNP-C	5 days	10 days	qPCR (no 16S)
Shen et al. [51]	HNP-C	Chitosan oligosaccharides + multi-strain probiotic mixture	Beagle dogs	Enrofloxacin + Metronidazole, 7 days	*n* = 8 × 2	Model vs. HNP-C	28 days	35 days	16S rRNA

AAD = Antibiotic-associated diarrhea, AB = Antibiotic, NR = Natural recovery, HNP = Herbal or natural product, HNP-C = Herbal–probiotic combination, PRO-only = Probiotic-only group. PC = Positive control (Inulin, FOS, GOS, etc.), NC = Normal control, L/M/H = Low/Medium/High dose.

## Data Availability

No new data were created or analyzed in this study. Data sharing is not applicable to this article.

## Supplementary Materials

The following supporting information can be downloaded at: https://www.mdpi.com/article/10.3390/ph19010064/s1, Table S1: PRISMA checklist; Table S2: Full database-specific search strategies for PubMed, EMBASE, Web of Science/Scopus, and CNKI.

## Abbreviations

The following abbreviations are used in this manuscript:

AAD	Antibiotic-associated diarrhea
SCFA/SCFAs	Short-chain fatty acid/short-chain fatty acids
HNP	Herbal/Natural Product
HNP-C	Herbal/Natural Product Combination
DGGE	Denaturing Gradient Gel Electrophoresis
PCoA	Principal Coordinates Analysis
NMDS	Non-metric Multidimensional Scaling
qPCR	Quantitative Polymerase Chain Reaction
GC–MS	Gas Chromatography–Mass Spectrometry
MUC2	Mucin 2
ZO-1	Zonula Occludens-1
OC-1/Occludin	Occludin (tight-junction protein)
TJ	Tight Junction
IgA/IgG/IgM	Immunoglobulin A/G/M
LPS	Lipopolysaccharide
DAO	Diamine Oxidase
FOXP3	Forkhead Box P3
IL-1β/IL-6/IL-10/IL-17A	Interleukin-1β/Interleukin-6/Interleukin-10/Interleukin-17A
TNF-α	Tumor Necrosis Factor-α
MAPK	Mitogen-Activated Protein Kinase
NF-κB	Nuclear Factor kappa-B
F/B ratio	Firmicutes-to-Bacteroidetes Ratio
PC	Positive Control
NC	Normal Control
L/M/H	Low/Medium/High dose
SIgA	Secretory Immunoglobulin A
Smad7/p-Smad3	Smad7/phosphorylated Smad3
CFU	Colony Forming Unit
