# Supplementary material for: Herbal and Natural Products for Antibiotic-Associated Diarrhea: A Systematic Review of Animal Studies Focusing on Molecular Microbiome and Barrier Outcomes
Source: Pharmaceuticals (Basel). 2025 Dec 29;19(1):64. doi: 10.3390/ph19010064 (PMC12845432; doi:10.3390/ph19010064)
Supplement: Supplementary file 1 [file pharmaceuticals-19-00064-s001.zip › pharmaceuticals-4040703-supplementary.pdf]

Table S2. Full database-specific search strategies for PubMed, EMBASE, Web of Science/Scopus, and CNKI.

**Pubmed**	**(("antibiotic associated diarrhea"[tiab] OR "antibiotic induced diarrhea"[tiab] OR AAD[tiab] OR "antibiotic-induced gut dysbiosis"[tiab]) AND (Herbal Medicine[Mesh] OR "Medicine, Chinese Traditional"[Mesh] OR Phytotherapy[Mesh] OR "Plant Extracts"[Mesh] OR "herbal medicine"[tiab] OR "traditional chinese medicine"[tiab] OR "botanical drug*"[tiab] OR "plant extract*"[tiab] OR Panax[Mesh] OR Ginseng[tiab] OR Zingiber[Mesh] OR Ginger[tiab] OR Glycyrrhiza[Mesh] OR Licorice[tiab] OR Astragalus[Mesh] OR Rheum[Mesh] OR Rhubarb[tiab] OR "Shengjiang Xiexin Decoction"[tiab] OR "Lizhong Tang"[tiab] OR "Banxia Xiexin Tang"[tiab] OR Synbiotics[Mesh] OR synbiotic*[tiab]) AND (Gastrointestinal Microbiome[Mesh] OR "gut microbiota"[tiab] OR microbiome[tiab] OR "intestinal flora"[tiab] OR "fecal microbiota"[tiab]) AND (animals[mh] NOT humans[mh]))**
**EMBASE**	('antibiotic associated diarrhea'/exp OR 'antibiotic induced diarrhea':ti,ab OR 'aad':ti,ab) AND ('gut microbiota'/exp OR 'intestinal microbiome'/exp OR 'fecal microbiota':ti,ab) AND ('herbal medicine'/exp OR 'phytotherapy'/exp OR 'plant extract'/exp OR 'traditional chinese medicine'/exp OR 'polysaccharide'/exp OR 'traditional medicine':ti,ab OR 'synbiotic'/exp OR synbiotic*:ti,ab OR 'probiotic'/exp OR probiotic*:ti,ab OR 'prebiotic'/exp OR prebiotic*:ti,ab) AND ('animal model'/exp OR 'animal experiment'/exp OR mouse:ti,ab OR mice:ti,ab OR rat:ti,ab OR rodent:ti,ab) NOT ('human'/exp OR 'human cell'/exp)
**WOS SCOPUS**	(TITLE-ABS-KEY("antibiotic-associated diarrhea" OR "antibiotic induced diarrhea" OR "AAD" OR "antimicrobial-associated diarrhea") AND TITLE-ABS-KEY("herbal medicine" OR "Chinese medicine" OR "traditional medicine" OR "phytotherapy" OR "plant extract" OR "polysaccharide" OR "natural product" OR "synbiotic") AND TITLE-ABS-KEY("gut microbiota" OR "gut microflora" OR "intestinal microbiota" OR "intestinal flora" OR "gut dysbiosis") AND TITLE-ABS-KEY("animal model" OR "mice" OR "mouse" OR "rats" OR "rodent"))
**CNKI**	The CNKI search used English-equivalent keywords corresponding to the original Chinese terms:<br>1. (“antibiotic-associated diarrhea” OR “antibiotic-induced diarrhea” OR “antimicrobial-associated diarrhea”)<br>2. (“herbal medicine” OR “natural product” OR “plant extract” OR “polysaccharide” OR “synbiotic”)<br>3. (“gut microbiota” OR “intestinal microbiota” OR “gut dysbiosis”)<br>4. (“animal model” OR “laboratory animals” OR “animal experiment” OR “mouse” OR “rat”)
